# Editorial: New approach methodologies for the assessment of cardiovascular diseases and drug induced cardiotoxicity

**DOI:** 10.3389/ftox.2026.1945177

**Published:** 2026-10-13

**Authors:** Fiona Chapman, Matthew Stevenson, Victoria Hutter, Paola Occhetta

**Affiliations:** 1 Imperial Brands PLC, Bristol, United Kingdom; 2 Department of Clinical, Pharmaceutical and Biological Science, University of Hertfordshire, Hatfield, United Kingdom; 3 Department of Electronics, Information and Bioengineering, Politecnico di Milano, Milan, Italy

**Keywords:** cardiotoxic adverse effect, cardiotoxicity, CVD (cardio vascular disease), *in vitro*, new approach methodologies

Cardiovascular disease (CVD) represents the largest cause of death globally, with a number of contributing mechanisms and risk factors (WHO, 2025). Alongside this, drug-induced cardiotoxicity is a leading cause of clinical adverse events and associated withdrawals from the market (Mamoshina et al., 2021). Cardiotoxic and CVD progression pathways are therefore of key research interest, to fill the gaps in disease biology and toxicity mechanisms, and support safety and regulatory testing strategies. Current chemical testing strategies are often limited in their scope in terms of capturing biological effect windows and toxicity progression, with assessment endpoints only covering limited, often late-stage events (Ladeira et al., 2026). Furthermore, strategies for testing complex mixtures, such as botanical extracts, are limited, and more generally, there is a need for greater efficiency in cardiotoxicity assessment, with a move towards higher-throughput models (Waidyanatha et al., 2024). Currently, drug testing in animals does not sufficiently detect potential adverse cardiotoxic events in humans, which also highlights the need for human-relevant models and endpoints (Van Norman, 2020).

This Research Topic, New Approach Methodologies for the assessment of Cardiovascular Diseases and Drug Induced Cardiotoxicity, is a Research Topic of articles relating to New Approach Methodologies (NAMs) that have the potential to address the current gaps in the assessment of cardiotoxicants and/or mechanisms of CVD development. The Research Topic presents *in vitro* human-relevant models, which provide both increased throughput and enable combined, holistic, toxicity-related endpoint assessment when applied in chemical/mixture cardiotoxicity testing. Further to this, by building Adverse Outcome Pathways (AOPs) related to cardiotoxicity and CVD, frameworks and biological targets have been identified to aid design of future assessments for those working within this area.

Assessment of novel small molecules for their cardiotoxic potential is an important step in their development pipeline. Szabo et al. carried out a comprehensive analysis of 17 small molecules with known cardiotoxic profiles using human induced pluripotent stem cell-derived cardiomyocytes (hiPSC-CMs), combining both morphological and electrophysiological endpoints. Their multiplexed testing approach highlighted that morphological analysis *in vitro* provided greater prediction of cardiotoxic potential than electrophysiology alone, however, combined, these endpoints acted as stronger predictors of clinical outcomes. The authors also delved deeper using RNA sequencing to attribute outcomes at the transcriptional level. The work outlines the promise of a multi-endpoint approach, using human-derived cells, moving towards the animal-free prediction of cardiotoxic outcomes, and discusses further directions this testing approach can take.

From known, single compounds, we move onto the effects of complex mixtures, which, due to the various combined chemical constituents, can present challenges in their cardiotoxicity assessment. Krzykwa et al. report a recommended testing approach of the Botanical Safety Consortium Cardiotoxicty Working Group for the assessment of botanical extracts. The group firstly considered the *in vitro* assays needed to provide insight into cardiotoxic effects, including electrophysiological measures, optical mapping approaches, metabolic and mechanical perturbation and standard safety pharmacology screening strategies. Through a selection of case studies, the group then discuss a range of botanical extracts, of varying toxicity and amounts of cardiotoxicological data available, with which the proposed assays will be applied in future work.

Cigarette smoking presents another scenario for exposure to complex chemical mixtures, and is a known risk factor in the development of CVD. Makena et al. propose an AOP for atherosclerotic plaque progression, with oxidative stress induced by cigarette smoke constituents as the Molecular Initiating Event. They identified seven key events from cellular to tissue level, with five rated as having high essentiality in the pathway. Through comprehensive mapping of these key events, this AOP provides insights into potential biomarkers and targets for investigation in smokers, or smoking-related research, with regards to atherosclerosis development potential. The work is also useful for other areas of research related to oxidative stress, highlighting its role in CVD development.

Ladeira et al. also highlighted the role of oxidative stress, amongst other key biological events, in their analysis of the interactions of multiple AOPs with relation to cardiotoxicity. Drawing upon the AOP-Wiki database, cardiotoxic outcomes were demonstrated to be the result of the interaction of distinct key events and organ systems. The authors used their analysis to build a live AOP network for cardiotoxicity events, which can be updated as new information becomes available. The network also provides a valuable reference point for the design of animal-free studies to gain mechanistic understanding in testing for potential chemical cardiotoxicity.

Combined, the four publications included in this Research Topic contribute some interesting and exciting insights for the advancement of assessment of chemical cardiotoxic potential and CVD development mechanisms. Although the applications of the works are varied, from pharmaceutical and botanical product safety, to longer-term CVD risk assessment, there are key learnings and themes which connect them. For example, through application of multiple endpoints, whether using a single hiPSC-CM cell model or utilising different studies to build AOP frameworks, the value of an in-depth and holistic mechanistic view is highlighted. Through building mechanistic networks and testing frameworks, the works also support the design of future studies within the field, for example, when novel compound cardiotoxicity characterization is required. These studies may then be targeted, using appropriate models, to ensure human relevance, tied to realistic clinical outcomes, and move away from the use of animals within such research where possible; targeted use of non-animal models may also increase throughput within cardiotoxicity screening assessments. This Research Topic provides a strong foundation for future studies, with a focus on NAMs, to build upon, and signposts key research areas for the advancement of the field of cardiotoxicity and CVD development assessment.
